# Detection of macular and peripheral ocular microvascular changes after carotid artery revascularization using widefield SS-OCTA

**DOI:** 10.3389/fmed.2025.1530867

**Published:** 2025-01-22

**Authors:** Ting Luo, Lu Wang, Li Zhang

**Affiliations:** ^1^Department of Ophthalmology, The Third People’s Hospital of Chengdu, Chengdu, China; ^2^Department of Ophthalmology, The Second People’s Hospital of Chengdu, Chengdu, China; ^3^Department of Ophthalmology, West China Hospital, Sichuan University, Chengdu, China

**Keywords:** carotid artery revascularization, SSOCTA, retinal microvasculature, foveal avascular zone (FAZ), carotid artery stenosis (CAS)

## Abstract

**Purpose:**

This study aims to investigate microvascular changes in the macular and peripheral regions, as well as alterations in the foveal avascular zone (FAZ) of the ipsilateral eye using widefield swept-source optical coherence tomography angiography (SS-OCTA) in patients with severe carotid artery stenosis (SCAS) after carotid artery revascularization (CAR).

**Design:**

This study employed a prospective study design.

**Methods:**

SCAS patients were examined using widefield SS-OCTA, which covered an area of 16 × 16 mm centered on the fovea. These examinations were conducted both before and after CAR to assess changes in retinal and choroidal blood flow in the macular and peripheral regions, as well as alterations in the characteristics of the FAZ.

**Results:**

A total of 56 patients and their corresponding 56 ipsilateral eyes were included in this study. In the macular area, a significant increase in the vessel density of the retinal superficial vascular complex (VDRSVC) was found, while a significant decrease was noted in the vessel density of the retinal deep vascular complex (VDRDVC) after CAR. Additionally, both the area and circumference of the FAZ decreased significantly after CAR. In the peripheral regions, significant increases were detected in both VDRSVC and the three-dimensional choroidal vascularity index (3D-CVI) post-CAR.

**Conclusion:**

The findings of this study demonstrated that CAR can improve perfusion in both the macular and peripheral fundus and lead to a decreased area and circumference of the FAZ in the ipsilateral eye. The observed decrease in the VDRDVC after CAR may be attributed to microvascular embolization and ischemia within the deep capillary plexus (DCP).

## Introduction

Carotid artery stenosis usually occurs at the bifurcation of the common carotid artery, involving the origin of the internal carotid artery (ICA) and the carotid bulb, which is one of the major causes of acute ischemic stroke (1, 2). The ophthalmic artery, which supplies the eye, is the first branch of the ICA. Thus, ICA stenosis can lead to decreased ocular blood flow. Severe stenosis of the carotid artery has been estimated to decrease perfusion pressure in the central retinal artery by approximately 50% (3). This chronic and progressive hypoperfusion of the eye may lead to global ocular ischemia and the development of ocular ischemic syndrome (OIS), potentially resulting in ocular pain, vision loss, and secondary glaucoma (3, 4). Previous studies have shown decreased macular microvascular perfusion in patients with severe carotid artery stenosis (SCAS), which improved after carotid artery revascularization (CAR) (5–11). It is common knowledge that the central macular (CM) is prioritized for perfusion to maintain visual function and is thus preserved under hypoperfusion conditions. Retinal hemorrhage, occurring in 80% of eyes affected by OIS, is the most frequent manifestation, with hemorrhage predominantly located in the mid-peripheral region outside the major vascular arcades instead of the macular region (4). To date, no studies have demonstrated changes in peripheral blood flow after CAR. Optical coherence tomography angiography (OCTA) is a non-invasive, time-efficient imaging modality that could provide detailed visualization of the perfusion of vascular networks in the ocular fundus. With advancements in widefield OCTA equipment, exploring the peripheral fundus microvasculature change has become possible. Additionally, the foveal avascular zone (FAZ), a specialized region in the CM area devoid of retinal vessels, has been studied in the context of ischemic retinal diseases such as diabetic retinopathy and retinal vein occlusion (12–14). However, changes in the FAZ after CAR have been less explored. This study aims to explore microvasculature changes in both the macular and peripheral regions and any alterations in the FAZ in the ipsilateral eye using widefield OCTA in SCAS patients undergoing CAR.

## Methods

### Study design and participants

This prospective, single-center, observational cohort study was conducted by the Department of Ophthalmology and Department of Neurology at West China Hospital, Sichuan University, Sichuan, China. The study was designed and performed following the ethical tenets outlined in the 1964 Declaration of Helsinki. It received approval from the Institute Ethics Committee of West China Hospital with verifiable consent (Approval Number 20231171). Consecutive patients were recruited from the hospitalized patients at the West China Hospital. All patients provided written informed consent before participating in this study. All recruited patients underwent a comprehensive clinical examination, which included assessments of best-corrected visual acuity (BCVA), slit lamp biomicroscopy, intraocular pressure (IOP), axial length, color fundus photography (CFP), and OCTA.

### Inclusion criteria

(1) Diagnosis of hemodynamically significant stenosis. The grade of carotid stenosis was evaluated using digital subtraction angiography (DSA) in accordance with the North America Symptomatic Carotid Endarterectomy Trial (NASCET) criteria. Significant stenosis is defined as having a stenosis ≥70% and (2) aged between 30 and 90 years.

### Exclusion criteria

(1) Presence of other ocular diseases such as glaucoma, diabetic retinopathy, or inflammatory retinopathy; (2) prior receipt of any fundus treatment, such as retinal laser, intravitreal injection, or vitrectomy; (3) axial length >26 mm or a mean spherical equivalent <−6 diopters; (4) severe postoperative complications, including central or branch retinal artery occlusion; (5) neurodegenerative or demyelinating diseases, such as Alzheimer’s disease, Parkinson’s disease, or multiple sclerosis; (6) increased IOP (>21 mmHg); (7) BCVA <0.2 (logMAR); and (8) poor quality of OCTA images (image quality ≤6) (15–18).

### Imaging protocols

Preoperatively, all participants underwent detailed ocular examination, including BCVA, IOP (TX-20, Canon, Tokyo), axial length (IOL Master Advanced Technology, Carl Zeiss, Meditec, Dublin, CA), slit lamp examination, CFP (CLARUS 500^™^ or Daytona, Optos), OCT, and OCTA (BM-400K BMizar, TowardPi Medical Technology, Beijing, China). Postoperatively (within 1 week), all participants will repeat the examination of IOP, slit lamp examination, CFP, OCT, and OCTA at the same time point of the day to avoid diurnal variations. Each type of examination should be conducted by the same appointed operator. All scans were adjusted based on axial length to prevent its influence on the results.

**Figure 1:**
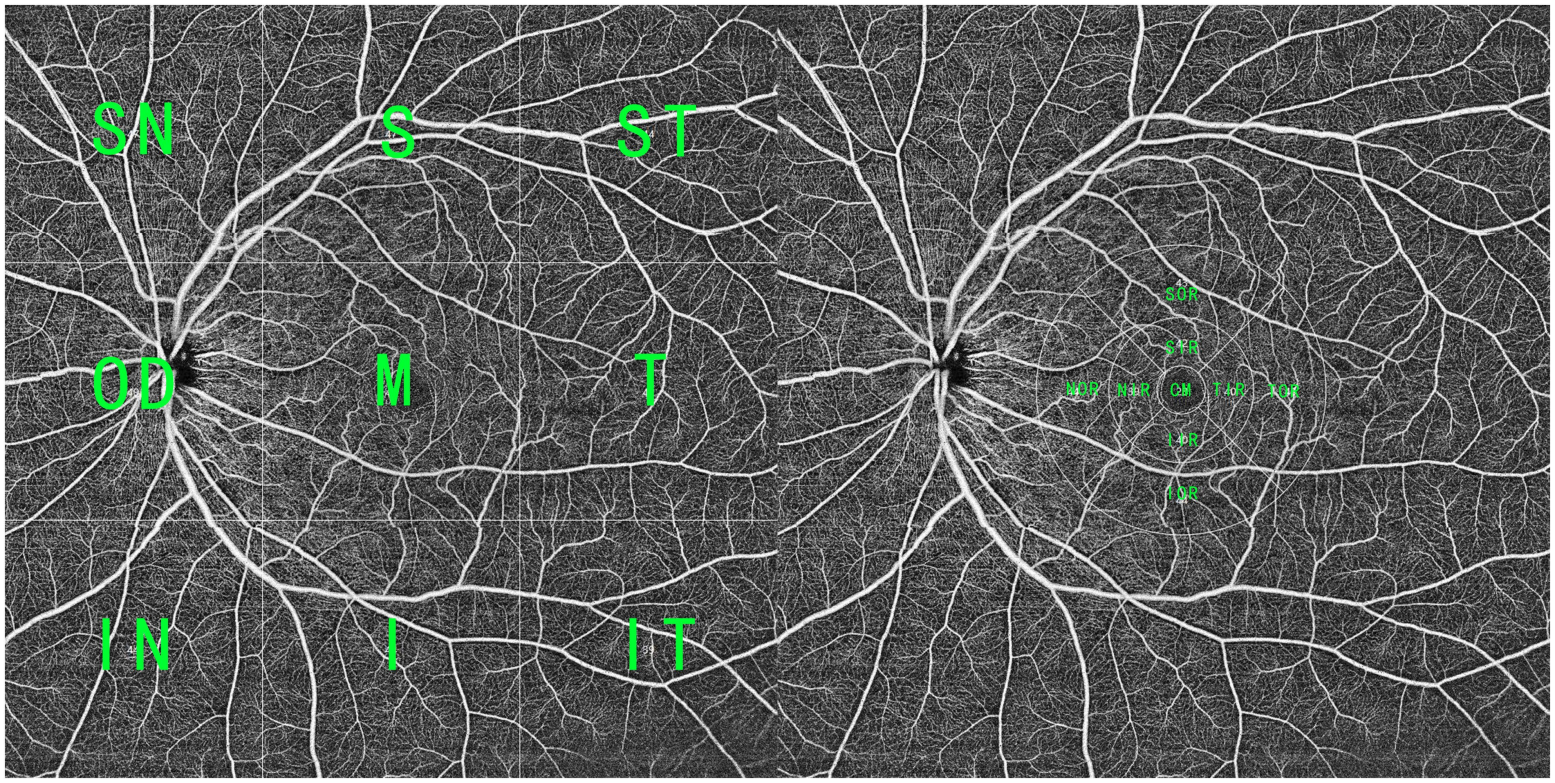
A widefield SS-OCTA scan within a range of 16 × 16 mm. (Left) The OCTA blood flow image was averagely divided into nine parts, and according to the adjacent relationship with the macular region, they were defined as superior temporal (1), superior (S), superior nasal (SN), temporal (T), macular (M), optic disc (OP), inferior temporal (IT), inferior, and inferior nasal (IN) regions. (Right) To analyze macular blood flow change, the macular region (CM with a bandwidth of 1 mm and two surrounding rings with a bandwidth of 3 mm) was divided into nine parts, including CM, superior of inner/outer ring (SIR/SOR), inferior of inner/outer ring (IIR/IOR), nasal of inner/outer ring (NIR/NOR), and temporal of inner/outer ring (TIR/TOR).

OCTA scans were obtained using a 400 kHz SS-OCTA instrument (BM-400K BMizar, TowardPi). It uses a swept-source VCSEL laser with a wavelength of 1,060 nm and a scanning rate of 400,000 A-scans per second, providing a transverse resolution of 10 μm and an in-depth resolution (optical) of 3.8 μm. This instrument has an A-scan depth of 6.0 mm in tissues (2,560 pixels). Two sequential B-scans were performed at each fixed position before proceeding to the next transverse location on the retina. The built-in software provides default segmentation layers, which include en face views of the superficial and deep inner retina plexus, outer retina plexus, and choriocapillaris, with artifacts minimized using volumetric projection artifact removal approaches. All of these segmentations were manually inspected and corrected as needed by two ophthalmologists (LZ and JWL) before any calculation. To capture a broader range of blood flow changes, a 16 × 16 mm volume was scanned. As shown in Figure 1, the 16 × 16 mm OCTA scans were divided into nine squares for analysis. According to the adjacent relationship with the macular region, they were defined as macular (M), superior temporal (ST), superior (S), superior nasal (SN), temporal (T), inferior (I), optic disc (OD), inferior temporal (IT), and inferior nasal (IN) regions. To analyze macular blood flow change, the macular region was further divided into nine subfields as the EDTRS grid (CM with a bandwidth of 1 mm and two surrounding rings with a bandwidth of 3 mm), including the CM, superior of inner/outer ring (SIR/SOR), inferior of inner/outer ring (IIR/IOR), nasal of inner/outer ring (NIR/NOR), and temporal of inner/outer ring (TIR/TOR).

To obtain FAZ characteristics, the images of the retinal blood flow layer were exported to an image processing and analysis software package (ImageJ; National Institutes of Health, version 1.46r). Each file was converted to an 8-bit image, and the scale was set to 320 pixels, representing 4.5 mm. The FAZ was manually outlined by two independent masked graders (TL and LZ), and the software automatically calculated the FAZ area and circumference.

### Measurement parameters

#### Retinal parameters

VDRSVC: Vessel density of the retinal superficial vascular complex.

VDRDVC: Vessel density of the retinal deep vascular complex.

#### Choroidal parameters

VDCML: Vessel density of choroidal middle and large vessels.

3D-CVI: Three-dimensional choroidal vascularity index. This is defined as the ratio of the choroidal vessel volume to the total choroidal volume measured with a three-dimensional algorithm, which reflects the volumetric choroidal vessel density.

#### FAZ parameters

FAZ area and FAZ circumference.

### Statistical analysis

All analyses were conducted using SPSS version 26 (SPSS, Inc., Chicago, IL, United States) and Microsoft Excel (version 16, Microsoft Corp, Redmond, WA, United States). The parametric data were reported as mean ± standard deviation, and the non-parametric data were reported as medians and interquartile ranges (IQR). Categorical variables were presented as numbers and percentages. A paired sample *t*-test or Wilcoxon signed-rank test was used to compare pre- and postoperative data based on the distribution. The paired *t*-test was used for normally distributed variables, and Wilcoxon tests were used for non-normally distributed variables. The chi-squared test, or Fisher’s exact test, was used for categorical variables. *p*-values of <0.05 were considered statistically significant.

## Results

### Demographic characteristics

In total, 31 patients declined postoperative examination and were excluded. A total of two patients with remote preoperative branch retinal artery occlusion, one patient with a macular hole, two patients with postoperative branch retinal artery occlusion, eight patients with epiretinal membrane, two patients with glaucoma, and 10 patients with poor-quality OCTA images were excluded. A total of 56 eyes from 56 SCAS patients with complete preoperative and postoperative data were included in this study between September 2023 and January 2024. Among them, 47 patients (83.9%) received carotid artery stenting, and 9 (16.1%) received carotid endarterectomy. The mean age was 65.34 ± 9.65 years. The axial length was 23.68 ± 0.82 and 23.70 ± 0.85 for the ipsilateral and contralateral eyes, respectively (Table 1).

**Table 1:** Demographic characteristics.

	SCAS patients
Age	65.34 ± 9.65
Sex	51 M (91.1%), 5 F (8.9%)
AL	
Ipsilateral eye	23.68 ± 0.82
Contralateral eye	23.70 ± 0.85
DM	23 (40.4%)
HT	37 (64.9%)
HG	10 (17.5%)
CAC	17 (29.8%)
Smoking	24 (42.9%)
Drinking	24 (42.9%)

SCAS, severe carotid artery stenosis; M, male; F, female; AL, axil length; DM, diabetes mellitus; HT, hypertension; HG, hyperlipidemia; CAC, coronary atherosclerotic cardiopathy.

### Macular microvascular change

Table 2 shows a comparison of preoperative and postoperative macular microvascular changes. The postoperative VDRSVC in the TOR [41.00 (39.00, 43.75)] showed a significant increase compared to the preoperative VDRSVC of the TOR [39.00 (36.25, 42.00), *p* < 0.001]. However, postoperative VDRDVC of the CM [29.00 (27.00, 34.75)], NIR [41.00 (39.00, 42.75)], and IIR [42.00 (39.25, 43.00)] have significantly decreased compared with preoperative VDRDVC of the CM [32.00 (27.00, 36.75), *p* = 0.003], NIR [41.50 (39.25, 44.00), *p* = 0.007], and IIR [43.00 (40.00, 44.00), *p* = 0.007]. No significant difference in VDCML has been found. However, postoperative 3D-CVI of TIR [38.00 (33.00, 41.75)], NIR [38.00 (33.00, 42.00)], TOR [34.00 (30.25, 36.00)], SOR [36.50 (34.00, 38.75)], NOR [33.00 (24.25, 40.75)] and IOR [35.00 (32.00, 38.75)] have significantly increased compared with preoperative 3D-CVI of TIR [36.00 (29.50, 38.00), *p* < 0.001], NIR [37.00 (31.25, 41.00), *p* = 0.007], TOR [34.00 (30.00, 35.75), *p* = 0.008], SOR [36.00 (33.00, 38.00), *p* = 0.025], NOR [33.00 (21.50, 39.00), *p* < 0.001] and IOR [35.00 (31.00, 38.00), *p* = 0.014].

**Table 2:** Comparison of macular OCTA parameters before and after the revascularization surgery.

	Preoperative	Postoperative	*p*-value
VDRSVC			
CM	32.00 [28.00, 34.75]	31.00 [29.00, 34.00]	0.905
TIR	39.00 [37.00, 41.75]	39.00 [37.00, 41.00]	1.000
SIR	41.00 [38.25, 43.00]	40.50 [37.00, 43.75]	0.523
NIR	39.00 [36.00, 41.00]	38.50 [36.00, 42.00]	0.632
IIR	40.00 [38.00, 42.75]	40.00 [37.00, 42.00]	0.899
TOR	39.00 [36.25, 42.00]	41.00 [39.00, 43.75]	<0.001^*^
SOR	44.00 [41.00, 45.00]	43.00 [41.00, 45.00]	0.858
NOR	43.00 [41.00, 45.00]	43.50 [40.00, 45.00]	0.553
IOR	43.00 [40.25, 45.75]	43.50 [41.00, 46.00]	0.785
VDRDVC			
CM	32.00 [27.00, 36.75]	29.00 [27.00, 34.75]	0.003^*^
TIR	42.00 [40.00, 44.00]	41.50 [39.25, 43.00]	0.774
SIR	43.00 [40.00, 44.00]	42.00 [40.00, 43.00]	0.348
NIR	41.50 [39.25, 44.00]	41.00 [39.00, 42.75]	0.007^*^
IIR	43.00 [40.00, 44.00]	42.00 [39.25, 43.00]	0.007^*^
TOR	42.00 [39.25, 43.00]	41.50 [40.00, 43.00]	0.532
SOR	42.00 [40.25, 44.00]	42.00 [40.25, 44.00]	0.553
NOR	42.50 [41.00, 44.00]	43.00 [42.00, 44.00]	0.487
IOR	42.00 [40.00, 44.00]	42.00 [40.00, 44.00]	0.900
VDCML			
CM	50.00 [47.00, 53.75]	50.00 [47.25, 56.00]	0.483
TIR	49.50 [47.00, 55.00]	50.50 [47.00, 56.00]	0.863
SIR	50.00 [47.25, 56.75]	50.00 [47.25, 56.75]	0.507
NIR	49.50 [47.00, 56.00]	50.50 [47.00, 57.75]	0.318
IIR	50.50 [46.50, 55.00]	50.00 [47.25, 56.00]	0.923
TOR	50.00 [48.25, 56.00]	50.00 [48.25, 56.75]	0.327
SOR	50.00 [49.00, 56.00]	51.00 [48.25, 57.00]	0.618
NOR	51.50 [48.00, 55.75]	52.00 [50.00, 56.75]	0.301
IOR	51.00 [49.00, 57.00]	51.00 [49.00, 57.00]	0.494
3D-CVI			
CM	37.50 [31.50, 40.75]	38.00 [33.00, 41.75]	0.179
TIR	36.00 [29.50, 38.00]	37.00 [32.25, 40.75]	<0.001^*^
SIR	36.00 [31.00, 40.00]	37.00 [32.00, 40.75]	0.255
NIR	37.00 [31.25, 41.00]	38.00 [33.00, 42.00]	0.007^*^
IIR	36.00 [30.25, 40.00]	37.00 [32.00, 40.00]	0.110
TOR	34.00 [30.00, 35.75]	34.00 [30.25, 36.00]	0.008^*^
SOR	36.00 [33.00, 38.00]	36.50 [34.00, 38.75]	0.025^*^
NOR	33.00 [21.50, 39.00]	33.00 [24.25, 40.75]	<0.001^*^
IOR	35.00 [31.00, 38.00]	35.00 [32.00, 38.75]	0.014^*^

VDRSVC, vessel density of the retinal superficial vascular complex; VDRDVC, vessel density of the retinal deep vascular complex; VDCML, vessel density of choroidal middle and large vessels; 3D-CVI, three-dimensional choroidal vascularity index; CM, central macular; TIR, temporal of inner ring; SIR, superior of inner ring; NIR, nasal of inner ring; IIR, inferior of inner ring; TOR, temporal of outer ring; SOR, superior of outer ring; NOR, nasal of outer ring; IOR, inferior of outer ring; ^*^p-values <0.05.

### FAZ change

**Figure 2:**
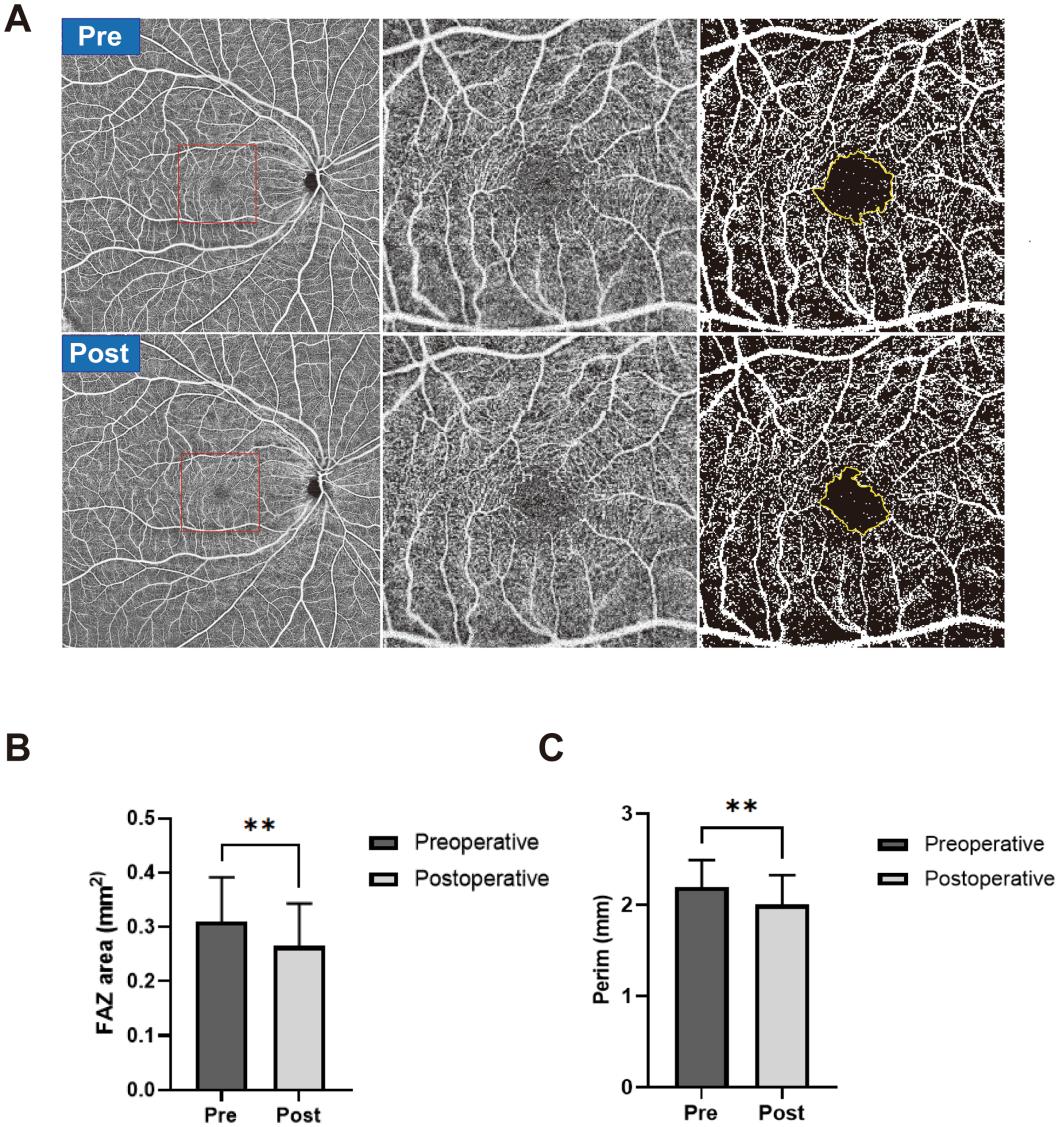
**(A)** A representative case of FAZ change in a severe CAS patient after CAR. The preoperative OCTA images are shown in the first row, and postoperative OCTA images are shown in the second row. Widefield OCTA blood flow images (left), magnified view (middle), and manually outlined yellow-colored FAZ boundary (right). **(B)** The FAZ area was manually outlined in the preoperative group (dark) and postoperative group (gray). **(C)** FAZ circumference was manually outlined in the preoperative group (dark) and postoperative group (gray). ^**^p < 0.01.

Figure 2 shows a representative case of FAZ change in a severe CAS patient after CAR and the results of FAZ area and circumference difference between the preoperative group and the postoperative group. The FAZ area was significantly smaller in the postoperative group (0.265 ± 0.078 mm^2^) than in the preoperative group (0.310 ± 0.082 mm^2^, *p* = 0.0036). The FAZ circumference was also significantly smaller in the postoperative group (2.003 ± 0.323 mm) than in the preoperative group (2.193 ± 0.300 mm, *p* = 0.0016).

### Peripheral fundus microvascular change

Table 3 shows the comparison of peripheral fundus microvascular changes between the preoperative group and the postoperative group. Postoperative VDRSVC of IT [39.50 (36.00, 42.00)] has significantly increased compared with preoperative VDRSVC of IT [38.00 (34.00, 40.00), *p* = 0.003]. Postoperative VDRDVC of IT [42.00 (39.25, 44.00)] has significantly increased compared with preoperative VDRDVC of IT [41.00 (37.00, 43.00), *p* = 0.014]. The 3D-CVI of OD, I, and IN increased after CAR (*p* < 0.05). No significant differences in microvascular changes were found in other regions of the peripheral fundus.

**Table 3:** Comparison of peripheral OCTA parameters before and after the revascularization surgery.

	Preoperative	Postoperative	*p*-value
VDRSVC			
ST	40.00 [38.00, 42.00]	41.00 [39.00, 42.75]	0.374
S	42.50 [40.00, 45.00]	44.00 [42.00, 45.00]	0.055
SN	44.00 [42.00, 45.00]	44.00 [41.00, 45.00]	0.701
T	39.50 [36.25, 42.00]	41.00 [38.00, 42.00]	0.081
OD	46.00 [45.00, 48.00]	47.00 [45.00, 48.00]	0.113
IT	38.00 [34.00, 40.00]	39.50 [36.00, 42.00]	0.003^*^
I	42.00 [38.00, 44.00]	42.00 [39.00, 44.00]	0.560
IN	41.00 [37.00, 43.00]	41.00 [39.00, 43.00]	0.158
VDRDVC			
ST	43.00 [41.00, 44.00]	43.00 [41.00, 45.00]	0.248
S	44.00 [42.00, 45.00]	44.00 [42.25, 45.00]	0.114
SN	43.00 [41.00, 45.00]	43.00 [41.00, 45.00]	0.930
T	42.00 [39.00, 43.75]	42.00 [40.25, 43.00]	0.131
OD	46.00 [43.00, 47.00]	45.50 [45.00, 47.00]	0.202
IT	41.00 [37.00, 43.00]	42.00 [39.25, 44.00]	0.014^*^
I	43.00 [41.25, 45.00]	43.00 [41.00, 45.00]	0.992
IN	43.00 [39.25, 45.00]	43.00 [41.00, 45.00]	0.360
VDCML			
ST	50.00 [47.00, 56.75]	51.00 [48.00, 57.00]	0.157
S	50.00 [49.00, 57.00]	50.00 [48.00, 57.00]	0.565
SN	51.00 [50.00, 58.00]	52.00 [50.00, 58.00]	0.917
T	51.00 [50.00, 57.75]	51.50 [50.00, 58.00]	0.853
OD	51.00 [49.00, 54.25]	51.00 [50.00, 55.50]	0.032^*^
IT	50.00 [48.00, 57.00]	50.50 [49.00, 57.00]	0.177
I	51.00 [50.00, 58.00]	51.50 [51.00, 58.00]	0.066
IN	53.00 [51.00, 55.00]	52.50 [51.00, 55.75]	0.127
3D-CVI			
ST	50.00 [47.00, 56.75]	51.00 [48.00, 57.00]	0.164
S	50.00 [49.00, 57.00]	50.00 [48.00, 57.00]	0.968
SN	51.00 [50.00, 58.00]	52.00 [50.00, 58.00]	0.234
T	51.00 [50.00, 57.75]	51.50 [50.00, 58.00]	0.467
OD	51.00 [49.00, 54.25]	51.00 [50.00, 55.50]	<0.001^*^
IT	50.00 [48.00, 57.00]	50.50 [49.00, 57.00]	0.054
I	51.00 [50.00, 58.00]	51.50 [51.00, 58.00]	0.005^*^
IN	53.00 [51.00, 55.00]	52.50 [51.00, 55.75]	0.001^*^

VDRSVC, vessel density of the retinal superficial vascular complex; VDRDVC, vessel density of the retinal deep vascular complex; VDCML, vessel density of choroidal middle and large vessels; 3D-CVI, three-dimensional choroidal vascularity index; ST, superior temporal; S, superior; SN, superior nasal; T, temporal; OD, optic disk; IT, inferior temporal; I, inferior; IN, inferior nasal; ^*^p-values <0.05.

## Discussion

This prospective study aims to investigate the macular and peripheral fundus microvascular changes after CAR. While earlier studies have explored the influence of CAR on macular microvascular change, few have explored the FAZ change and microvascular changes of the peripheral fundus.

According to the results, VDRSVC of TOR has greatly improved after CAR, which was consistent with previous studies (5–10), indicating improved macular retinal blood supply with the opening of the occluded carotid artery.

Change of choroidal thickness has been reported with different results in the literature (7, 19–25), probably due to its variability, as it could be influenced by other factors such as age, sex, axial length, and diurnal fluctuations in choroidal thickness measurements (26). Some systemic conditions or drug therapies can also impact the results of choroidal thickness measurements. In contrast, 3D-CVI, which takes into account both the vascular and interstitial components of the choroid within three-dimensional maps, was a more robust and reliable biomarker with less variability (27). However, reports have identified the impact of image brightness on the measurement of CVI (28). In this study, we found that the 3D-CVI of TIR, NIR, TOR, SOR, NOR, and IOR had greatly improved after CAR, which may indicate improved choroidal blood perfusion after CAR.

However, decreased VDRDVC in the macular regions of CM, NIR, and IIR have been found in our results, which seems to contradict previous findings. Karapapak et al. (29) reported significantly improved vessel density of the deep capillary plexus (DCP) in both ipsilateral and contralateral eyes after carotid stenting. While Cao et al. (30) reported no changes in the deep vascular complex (DVC) following carotid stenting, the retinal DVC includes the intermediate capillary plexus (ICP) and the DCP. A significant proportion of the retinal arterial flow primarily passes through the superficial capillary plexus (SCP), while the DVC receives comparatively lower blood inflow (31, 32). Meanwhile, the middle retina may need more oxygen supply with its more significant metabolic activity than the inner retina (33). These have made the DVC excessively vulnerable to ischemia. Ischemia of the DVC has been described as paracentral acute middle maculopathy (PAMM), which manifests as a hyperreflective lesion at the level of the inner nuclear layer (34). Joseph et al. (35) have reported a case of PAMM following carotid stenting and attributed this to small particles that may have obstructed the pre-capillary arteriolar. It has also been demonstrated that the risk of periprocedural stroke or death was higher following stenting when compared with endarterectomy, which could be attributed to an increase in minor, non-disabling strokes (36). As for the eye, different studies reported that retinal embolization varies between 15 and 16.9% following carotid stenting and 1–4.9% following endarterectomy (37–40). Recently, our publication demonstrated a phenomenon of postoperative ipsilateral choriocapillaris flow voids (PICCVF), which indicated microembolization of the choriocapillaris following stenting (41). Combined with findings within the present study, these have strengthened our hypothesis that microembolization exists in the retinal microvasculature more frequently than we have seen clinically.

The FAZ is a special capillary-free region that forms a ring of interconnecting capillaries at the margin of the fovea. Changes in its morphology and perifoveal capillary density may reflect the degree of macular ischemia and are closely related to retinal vascular diseases, such as diabetic retinopathy and retinal vein occlusion (12–14). In this study, we found significantly decreased FAZ area and circumference in the postoperative group compared with the preoperative group following CAR, which may reflect improved macular perfusion and relief of macular ischemia after CAR.

There are several limitations in this study. First, we only detected short-term microvascular changes after CAR. Longitudinal studies on long-term microvascular changes may help us better understand the influence of CAR on the eye. Second, a variation of 2 to 7 days in the timing of the postoperative examination may have affected the results. Finally, the sample size is relatively small.

Future studies may explore the imaging features of OCTA and color fundus photography after CAR and their association with ocular microvascular hemodynamics and cerebral hemodynamics. With the development of ultra-widefield OCTA devices, the microvasculature in more peripheral regions of the fundus is now available to be analyzed, which may provide additional insights into the effects of CAR on ocular perfusion.

In conclusion, our study has provided detailed and comprehensive results on macular and peripheral microvascular changes in the fundus after CAR, utilizing widefield SS-OCTA. The findings of this study also demonstrated that CAR could improve both macular and peripheral fundus perfusion and reduce the FAZ area and circumference of the FAZ in the ipsilateral eye. The observed decrease in VDRDVC after CAR may result from microvascular embolization and ischemia in the DCP.

## Data Availability

The raw data supporting the conclusions of this article will be made available by the authors, without undue reservation.

## Glossary

CAR: Carotid artery revascularization
SCAS: Severe carotid artery stenosis
SS-OCTA: Swept-source optical coherence tomography angiography
FAZ: Foveal avascular zone
BCVA: Best-corrected visual acuity
IOP: Intraocular pressure
CFP: Color fundus photography
VDRSVC: Vessel density of retinal superficial vascular complex
VDRDVC: Vessel density of retinal deep vascular complex
3D-CVI: Three-dimensional choroidal vascularity index
ICA: Internal carotid artery
OIS: Ocular ischemic syndrome
CM: Central macula
SIR: Superior of inner ring
SOR: Superior of the outer ring
IIR: Inferior of inner ring
IOR: Inferior of outer ring
NIR: Nasal of inner ring
NOR: Nasal of outer ring
TIR: Temporal of inner ring
TOR: Temporal of outer ring
ICP: Intermediate capillary plexus
DCP: Deep capillary plexus
SCP: Superficial capillary plexus
PAMM: Paracentral acute middle maculopathy

## References

[1] Cheng SF Brown MM Simister RJ Richards T. Contemporary prevalence of carotid stenosis in patients presenting with ischaemic stroke. Br J Surg. (2019) 106:872–8. doi: 10.1002/bjs.11136, PMID: 30938840

[2] Jusufovic M Skagen K Krohg-Sørensen K Skjelland M. Current medical and surgical stroke prevention therapies for patients with carotid artery stenosis. Curr Neurovasc Res. (2019) 16:96–103. doi: 10.2174/1567202616666190131162811, PMID: 30706783

[3] Terelak-Borys B Skonieczna K Grabska-Liberek I. Ocular ischemic syndrome—a systematic review. Med Sci Monit. (2012) 18:RA138. doi: 10.12659/MSM.883260, PMID: 22847215 PMC3560693

[4] Mendrinos E Machinis TG Pournaras CJ. Ocular ischemic syndrome. Surv Ophthalmol. (2010) 55:2–34. doi: 10.1016/j.survophthal.2009.02.024, PMID: 19833366

[5] Batu Oto B Kılıçarslan O Kayadibi Y Yılmaz Çebi A Adaletli İ Yıldırım SR. Retinal microvascular changes in internal carotid artery stenosis. J Clin Med. (2023) 12:6014. doi: 10.3390/jcm12186014, PMID: 37762953 PMC10531601

[6] Liu J Wan J Kwapong WR Tao W Ye C Liu M. Retinal microvasculature and cerebral hemodynamics in patients with internal carotid artery stenosis. BMC Neurol. (2022) 22:386. doi: 10.1186/s12883-022-02908-7, PMID: 36229769 PMC9559035

[7] Akca Bayar S Kayaarası Öztürker Z Pınarcı EY Ercan ZE Akay HT Yılmaz G. Structural analysis of the retina and choroid before and after carotid artery surgery. Curr Eye Res. (2020) 45:496–503. doi: 10.1080/02713683.2019.1666994, PMID: 31507205

[8] Wu DH Wu LT Wang YL Wang JL. Changes of retinal structure and function in patients with internal carotid artery stenosis. BMC Ophthalmol. (2022) 22:123. doi: 10.1186/s12886-022-02345-7, PMID: 35287632 PMC8922770

[9] Xu Q Sun H Yi Q. Association between retinal microvascular metrics using optical coherence tomography angiography and carotid artery stenosis in a Chinese cohort. Front Physiol. (2022) 13:824646. doi: 10.3389/fphys.2022.824646, PMID: 35721537 PMC9204184

[10] Liu X Yang B Tian Y Ma S Zhong J. Quantitative assessment of retinal vessel density and thickness changes in internal carotid artery stenosis patients using optical coherence tomography angiography. Photodiagn Photodyn Ther. (2022) 39:103006. doi: 10.1016/j.pdpdt.2022.103006, PMID: 35835327

[11] Ma F Su J Shang Q Ma J Zhang T Wang X. Changes in ocular hemodynamics after carotid artery angioplasty and stenting (CAAS) in patients with different severity of ocular ischemic syndrome. Curr Eye Res. (2018) 43:266–72. doi: 10.1080/02713683.2017.1390771, PMID: 29135355

[12] Liu J He Y Kong L Yang D Lu N Yu Y. Study of foveal avascular zone growth in individuals with mild diabetic retinopathy by optical coherence tomography. Invest Ophthalmol Vis Sci. (2023) 64:21. doi: 10.1167/iovs.64.12.21, PMID: 37698529 PMC10501493

[13] Waheed NK Rosen RB Jia Y Munk MR Huang D Fawzi A. Optical coherence tomography angiography in diabetic retinopathy. Prog Retin Eye Res. (2023) 97:101206. doi: 10.1016/j.preteyeres.2023.101206, PMID: 37499857 PMC11268430

[14] Balaratnasingam C Inoue M Ahn S McCann J Dhrami-Gavazi E Yannuzzi LA. Visual acuity is correlated with the area of the foveal avascular zone in diabetic retinopathy and retinal vein occlusion. Ophthalmology. (2016) 123:2352–67. doi: 10.1016/j.ophtha.2016.07.008, PMID: 27523615

[15] Gioia M De Bernardo M Rosa N Capasso L. Comment on: choroidal structural analysis in Alzheimer disease, mild cognitive impairment, and cognitively healthy controls. Am J Ophthalmol. (2021) 225:207–8. doi: 10.1016/j.ajo.2020.11.025, PMID: 33444634

[16] Picillo M Salerno G Tepedino MF Abate F Cuoco S Gioia M. Retinal thinning in progressive supranuclear palsy: differences with healthy controls and correlation with clinical variables. Neurol Sci. (2022) 43:4803–9. doi: 10.1007/s10072-022-06061-4, PMID: 35411501 PMC9349141

[17] De Bernardo M Salerno G Gioia M Capasso L Russillo M Picillo M. Intraocular pressure and choroidal thickness postural changes in multiple system atrophy and Parkinson’s disease. Sci Rep. (2021) 11:8936. doi: 10.1038/s41598-021-88250-3, PMID: 33903644 PMC8076309

[18] De Bernardo M Diana F Gioia M De Luca M Tepedino MF Pellecchia MT. The correlation between retinal and choroidal thickness with age-related white matter hyperintensities in progressive supranuclear palsy. J Clin Med. (2023) 12:6671. doi: 10.3390/jcm12206671, PMID: 37892809 PMC10607459

[19] Lareyre F Nguyen E Raffort J Carboni J Doyen J Hassen-Khodja R. Changes in ocular subfoveal choroidal thickness after carotid endarterectomy using enhanced depth imaging optical coherence tomography: a pilot study. Angiology. (2018) 69:574–81. doi: 10.1177/0003319717737223, PMID: 29082746

[20] Krytkowska E Masiuk M Kawa MP Grabowicz A Rynio P Kazimierczak A. Impact of carotid endarterectomy on choroidal thickness and volume in enhanced depth optical coherence tomography imaging. J Ophthalmol. (2020) 2020:8326207. doi: 10.1155/2020/8326207 32280535 PMC7125458

[21] Biberoglu E Eraslan M Midi I Baltacioglu F Bitargil M. Ocular blood flow and choroidal thickness changes after carotid artery stenting. Arq Bras Oftalmol. (2020) 83:417–23. doi: 10.5935/0004-2749.20200081, PMID: 33084820 PMC12289278

[22] Durusoy GK Gumus G Onay M Altay CM Binboga AB. Early choroidal structure and choroidal vascularity index change after carotid stenting. Photodiagn Photodyn Ther. (2022) 38:102748. doi: 10.1016/j.pdpdt.2022.102748, PMID: 35134537

[23] Zhang Y Zhou SW Noam N Rabinovitch D Bar D Yousif BS. Influence of carotid endarterectomy on choroidal perfusion: the INFLATE study. Ophthalmol Retina. (2024) 8:62–71. doi: 10.1016/j.oret.2023.07.026, PMID: 37531996

[24] Rabina G Barequet D Mimouni M Rabinovitch Y Wolf Y Barak A. Carotid artery endarterectomy effect on choroidal thickness: one-year follow-up. J Ophthalmol. (2018) 2018:8324093. doi: 10.1155/2018/8324093 30662767 PMC6312583

[25] Ala-Kauhaluoma M Koskinen SM Silvennoinen H Vikatmaa P Nuotio K Ijäs P. Subfoveal choroidal thickness in ipsi- and contralateral eyes of patients with carotid stenosis before and after carotid endarterectomy: a prospective study. Acta Ophthalmol. (2021) 99:545–52. doi: 10.1111/aos.14648, PMID: 33354923

[26] Brown JS Flitcroft DI Ying GS Francis EL Schmid GF Quinn GE. In vivo human choroidal thickness measurements: evidence for diurnal fluctuations. Invest Ophthalmol Vis Sci. (2009) 50:5–12. doi: 10.1167/iovs.08-1779, PMID: 18719079 PMC4112498

[27] Liu F Ye Y Yang W Wang J Xu Y Zhao Y. Quantitative evaluation of the topographical maps of three-dimensional choroidal vascularity index in children with different degrees of myopia. Invest Ophthalmol Vis Sci. (2024) 65:14. doi: 10.1167/iovs.65.3.14, PMID: 38466287 PMC10929746

[28] Rosa N Gioia M Orlando R De Luca M D’Aniello E Fioretto I. Impact of brightness on choroidal vascularity index. J Clin Med. (2024) 13:1020. doi: 10.3390/jcm13041020, PMID: 38398333 PMC10889141

[29] Karapapak M Ermis S Aksöz Bolat P Cingöz M Erdim Ç Özal E. Changes in retinal vascular density measured by optical coherence tomography angiography in patients with carotid artery stenosis after carotid artery stenting and angioplasty. Int Ophthalmol. (2024) 44:128. doi: 10.1007/s10792-024-03069-x, PMID: 38467951

[30] Cao L Wu J Wang H Kwapong WR Yan Y Wan J. Influence of carotid artery stenting on the retina and choroid. Transl Vis Sci Technol. (2024) 13:5. doi: 10.1167/tvst.13.8.5, PMID: 39093294 PMC11305422

[31] An D Yu P Freund KB Yu DY Balaratnasingam C. Three-dimensional characterization of the normal human parafoveal microvasculature using structural criteria and high-resolution confocal microscopy. Invest Ophthalmol Vis Sci. (2020) 61:3. doi: 10.1167/iovs.61.10.3, PMID: 32749461 PMC7443114

[32] Abtahi SH Nourinia R Mazloumi M Nouri H Arevalo JF Ahmadieh H. Retinal ischemic cascade: new insights into the pathophysiology and imaging findings. Surv Ophthalmol. (2023) 68:380–7. doi: 10.1016/j.survophthal.2022.11.009, PMID: 36464134

[33] Linsenmeier RA Zhang HF. Retinal oxygen: from animals to humans. Prog Retin Eye Res. (2017) 58:115–51. doi: 10.1016/j.preteyeres.2017.01.003, PMID: 28109737 PMC5441959

[34] Scharf J Freund KB Sadda SV Sarraf D. Paracentral acute middle maculopathy and the organization of the retinal capillary plexuses. Prog Retin Eye Res. (2021) 81:100884. doi: 10.1016/j.preteyeres.2020.100884, PMID: 32783959

[35] Alsberge JB McDonald HR. Paracentral acute middle maculopathy following internal carotid artery stenting. Am J Ophthalmol Case Rep. (2022) 28:101704. doi: 10.1016/j.ajoc.2022.101704, PMID: 36160271 PMC9493289

[36] Müller MD Lyrer P Brown MM Bonati LH Cochrane Stroke Group. Carotid artery stenting versus endarterectomy for treatment of carotid artery stenosis. Cochrane Database Syst Rev. (2020) 2020:CD000515. doi: 10.1002/14651858.CD000515.pub5, PMID: 32096559 PMC7041119

[37] Gaunt ME Rimmer T Smith JL Bell PRF Naylor AR. The effect of perioperative embolisation on visual function in patients undergoing carotid endarterectomy. Eur J Vasc Endovasc Surg. (1998) 16:231–7. doi: 10.1016/S1078-5884(98)80225-6, PMID: 9787305

[38] Vos JA van Werkum MH Bistervels JHGM Ackerstaff RGA Tromp SC van den Berg JC. Retinal embolization during carotid angioplasty and stenting: periprocedural data and follow-up. Cardiovasc Intervent Radiol. (2010) 33:714–9. doi: 10.1007/s00270-009-9775-4, PMID: 20033690

[39] Wilentz JR Chati Z Krafft V Amor M. Retinal embolization during carotid angioplasty and stenting: mechanisms and role of cerebral protection systems. Catheter Cardiovasc Interv. (2002) 56:320–7. doi: 10.1002/ccd.10232 12112883

[40] Song G Sun R Chen YF Ma Y Wang YB Jiao LQ. Retinal embolization after carotid endarterectomy and stenting for carotid artery stenosis. J Clin Neurosci. (2015) 22:1298–302. doi: 10.1016/j.jocn.2015.01.033, PMID: 25986182

[41] Zhang L Liu JW Tang QQ Lei CY Lin X Gao S. Decreased choriocapillaris vessel density in the ipsilateral eye after carotid artery revascularization detected by widefield swept-source OCT angiography. Ophthalmol Sci. (2025) 5:100654. doi: 10.1016/j.xops.2024.100654 39811266 PMC11730213

